# Aptenodytes forsteri optimization algorithm for low-carbon logistics network under demand uncertainty

**DOI:** 10.1371/journal.pone.0297223

**Published:** 2024-01-29

**Authors:** Yuhua Zhu, Xiang Fan, Chuanzhong Yin

**Affiliations:** College of Transport and Communications, Shanghai Maritime University, Shanghai, China; University of Victoria / Universiti Teknologi Malaysia /, CANADA

## Abstract

As China’s "double carbon" goal continues to advance, logistics as a key area of carbon emissions and low-carbon logistics center site selection are key links in the process. However, existing studies on logistics center location often ignore the impact of demand uncertainty, which leads to a waste of resources in the planning and construction processes. We take logistics cost and carbon emission as the objectives, and the multi-objective site selection model established based on stochastic programming theory takes demand uncertainty as a stochastic constraint. We transform the stochastic constraint model into a 0–1 mixed integer multi-objective planning model by utilizing the idea of equivalence transformation. The Aptenodytes Forsteri Optimization (AFO) algorithm is combined with the Ideal Point Method to solve the model, and the algorithm is compared with the Particle Swarm Optimization (PSO), Differential Evolutionary (DE), Tabu Search (TS), Sparrow Search (SS) algorithms, and the exact solver Linear Interactive and General Optimizer (LINGO). The examples verify the validity of the models and algorithms, with an average reduction of 6.2% and 3.6% in logistics costs and carbon emissions in the case of demand determination, and at the confidence level of 0.9 under demand uncertainty, both logistics costs and carbon emissions are decreased to varying degrees. This study provides a new research idea for the low-carbon logistics location problem under demand uncertainty, which helps to promote the transformation of the logistics industry to low-carbon and high-efficiency.

## 1.Introduction

In recent years, with the carbon peak and carbon neutral strategic goals proposed, low-carbon logistics has become more and more important in the national green development strategy. Logistics center location is an important element of logistics facilities and network planning, and the decision of logistics center location is related to sustainable development and is the key to achieving green and low-carbon goals. The problem of logistics center location usually requires two types of decisions: one to determine the number and location of logistics centers, and another to design the distribution scheme from logistics centers to each demand point.

In practice cargo demand is subject to external influences and is usually uncertain, Snyder [1] and Unnikrishnan [2] pointed out that ignoring uncertainty in cargo demand can lead to sub-optimal decisions in facility siting, supply chain and freight optimization. Musavi [3] pointed out that the biggest difficulty in logistics site selection under uncertain demand is how to make the optimal decision under an uncertain demand. Schuster Puga [4] pointed out in his study that demand uncertainty will significantly affect the decision of logistics location. The results of the above study show that the uncertainty of the demand for goods in the logistics site selection process cannot be ignored.

Therefore, we integrate the demand uncertainty associated with decision making in the strategic planning stage of facility siting, study the carbon emissions of logistics location from the perspective of vehicle fuel consumption, and propose an optimization model for logistics location under demand uncertainty to construct a multi-objective mathematical model with the objectives of minimum logistics cost and minimum carbon emissions. For the uncertainty of cargo demand, this paper uses the stochastic programming idea to transform the uncertain stochastic programming into a meaningful opportunity-constrained model. Since the stochastic programming approach is based on the important assumption that the probability distributions of uncertain parameters must be known or estimated, by reviewing and summarizing the references [5–15] related to supply chain demand uncertainty, as shown in Table 1, the relevant scholars assume the distribution of cargo demand as normal when studying cargo demand uncertainty, so this paper assumes the cargo demand as normal with reference to the previous studies.

**Table 1:** Literature related to demand probability distribution assumptions.

Paper	Probability distribution	Paper	Probability distribution
Fukasawa [5]	normal distributions	Nikzad [11]	normal distributions
Tokgöz [6]	normal distributions	Shahabi [12]	normal distributions
Saberi [7]	normal distributions	Wang [13]	Lognormal distribution
Alvarez [8]	normal distributions uniform distribution gamma distributions	Park [14]	normal distributions
Basciftci [9]	normal distributions	Mantrala [15]	normal distributions
Zandkarimkhani [10]	normal distributions	——	——

The low carbon logistics center location problem is usually solved by a mathematical model, and the logistics center site location mathematical model problem belongs to the complex combinatorial problem [16]. It has been shown to be NP-hard, meaning that there is no known algorithm that can provide exact solutions for all instances in polynomial time. Therefore, the main solution methods are mathematical planner solutions and metaheuristic algorithms [17].

Mathematical planners, including LINGO, CPLEX software, etc., have significant advantages in solving small-scale optimization problems; however, when the optimization problem is large in size, the mathematical planners are unable to obtain the appropriate solution in the specified time. On the other hand, metaheuristic algorithms such as genetic algorithm (GA) [18], PSO algorithm [19], hybrid clustered ant colony algorithm (ACO K-means) [20], and sorting-based heuristics [21] are prone to local optimality.

The "no-free lunch" theory states that no optimization algorithm is optimal for all problems. Each algorithm has a specific application area and may be superior to others on some problems. Therefore, considering these limitations, this paper adopts the AFO algorithm proposed by Yang [22] to solve the logistics center siting problem. Compared with other methods, the AFO algorithm exhibits a faster convergence speed in the process of searching for the optimal solution and incorporates a policy to prevent premature convergence, thus effectively avoiding the risk of falling into a local optimum (see Section 4.1.2 for details).

In this case, we consider logistics costs and carbon emissions and use stochastic programming ideas to build a multi-objective mathematical model with uncertain demand as the objective of the stochastic constraint. design an algorithm to combine the ideal point method and the AFO algorithm to solve the logistics site location model, with supermarket logistics site location as an example, and verify the effectiveness of the proposed model and algorithm. We validate the effectiveness of the proposed model as well as the algorithm by comparing it with conventional algorithms (e.g., PSO, DE, TS, and SS) and mathematical planners (LINGO). The main contributions of this paper are as follows:

1. We design the AFO-ideal point method algorithm to solve the demand uncertainty low-carbon logistics center siting problem, which provides a new research idea for research in this field and helps to promote the logistics industry to low-carbon and high-efficiency transformation.
2. We conduct a sensitivity analysis of freight rates, confidence levels, and standard deviations based on actual data to deeply analyze the impact of these parameters on logistics site selection options.
3. We provide a basis for decision-making and recommendations on how to avoid demand uncertainty disturbances.

The remainder of this paper is organized as follows: In Section 2, we review the relevant research literature. In Section 3, we describe the problem and give the mathematical formulation for solving it, followed by the solution algorithm in Section 4. In Section 5, we apply the proposed method and solution algorithm to the supermarket logistics center location to verify the validity of the model and algorithm and perform a sensitivity analysis on the influencing factors. Finally, Section 6 draws out our conclusions and future research directions.

## 2. Literature review

In this section, we first review the research progress of the green supply chain, then provide an overview of the related research on logistics center location under demand uncertainty, and finally point out the innovation of this study.

Regarding the research on the location of green logistics centers, Wang [23] studied the relationship between logistics costs and the environment and found that supply chain networks with higher capacity have lower total costs and lower carbon dioxide emissions. Yin [24] developed a multimodal supply chain optimization model for regional hubs with spatio-temporal constraints based on the regional integration perspective. Chaabane [25] studied and designed a closed-loop supply chain model that uses a life-cycle assessment (LCA) approach to demonstrate effective strategies for carbon management in an emissions trading scenario. Diabat [26] studied the green supply chain site-inventory problem, modeling it considering carbon allowance policies; Rezaee [27] proposed a carbon trading scenario based on a scenario-based carbon price based carbon trading scenario, proposed a two-stage stochastic optimization model to design a green supply chain network. Seydanlou [28] designed a sustainable closed-loop supply chain model based on sustainable supply chain management practices, considering economic, environmental, and social sustainability.

This paper investigates the problem of locating low-carbon logistics centers under uncertain demand, and Table 1 shows the research on uncertain demand and low-carbon logistics location in recent years. Sundarakani [29] studied the logistics location problem under uncertain demand using a robust optimization approach, converting carbon emissions to carbon costs, and solving it using LINDO API software. Yin [30] studied the p-hub low carbon location problem under uncertain demand using robust optimization method to convert carbon emissions into carbon cost and solved it using Cplex software. Wang [31] studied the supply chain-site-inventory problem under uncertain demand utilizing a stochastic programming approach, converting carbon emissions into carbon costs, and solving it using metaheuristic algorithms; Mechouar [32] studied the impact of carbon tax policies on siting options under demand determination, converted carbon emissions into carbon costs to construct a siting model with the minimum total cost, and solved it using the Generalized Reduced Gradient (GRG) algorithm. Wang [33] used a stochastic programming approach to consider the supply chain location problem under uncertain demand, developed a multi-objective model for logistics costs and carbon emissions, and solved the Pareto solution set utilizing a metaheuristic algorithm. Shen [34] studied the supply chain location problem under uncertain demand utilizing stochastic programming, constructed a multi-objective model of logistics cost and carbon emission, converted the multi-objective into a single objective using a linear weighting method, and then solved it using an improved genetic algorithm. Boskabadi [35] used a fuzzy programming approach to study the retail supply chain location problem under uncertain demand, developed a multi-objective model for logistics costs and carbon emissions, and solved the Pareto solution set utilizing a metaheuristic algorithm. Ali [36] established establish multi-objective optimization model for a closed-loop supply chain (CLSC) network considering economic, environmental, and social criteria and complex constraints. The stochastic programming idea was utilized to deal with the uncertain parameters, and a new Lagrangian relaxation algorithm was introduced to solve the problem effectively.

According to Table 2, the existing literature does not fully consider carbon emission sources when studying the multi-objective low-carbon siting problem and instead concentrates on transportation carbon emissions from the emission coefficient of transportation mode carbon, with limited investigation of the relationship between carbon emissions and fuel consumption, and with less consideration of cargo transshipment carbon emissions. The solution to carbon emissions is mainly to convert carbon emissions into carbon costs in order to construct a total cost minimization mathematical model and a multi-objective mathematical model with carbon emission as the objective function. The multi-objective mathematical model is mainly based on the linear weighting method and solving the Pareto solution set. This paper utilizes the ideal point method to solve multi-objective planning problems. The ideal point method usually has high computational efficiency in solving multi-objective planning, and it can find the optimal solution in a short time through effective algorithms and computational techniques. The ideal point method is based on mathematical models and objective data for calculation, which reduces the influence of personal preferences and subjective judgments of decision makers on the final results.

**Table 2:** Demand uncertainty low carbon logistics center site location literature.

Paper	Problem		Demand		Carbon		Uncertain parameter solution	Model solving methods
	Location	Carbon emission	Deterministic	Uncertain	Cost of Carbon	Carbon emisson		
Sundarakani [29]	✔	✔		✔	✔		Robust optimization	LINDO API
Yin [30]	✔	✔			✔		Robust optimization	Cplex
Wang [31]	✔	✔		✔	✔		Stochastic Programming	Metaheuristic algorithms
Mechouar [32]	✔	✔	✔			✔	——	GRG algorithms
Wang [33]	✔	✔		✔		✔	Stochastic Programming	Metaheuristic algorithm-Pareto solution set
Shen [34]	✔	✔		✔		✔	Stochastic Programming	Metaheuristic Algorithm—Linear Weighted
Boskabadi [35]	✔	✔	✔	✔		✔	fuzzy programming	Metaheuristic algorithm-Pareto solution set
Ali, S. M [36]	✔	✔		✔		✔	Stochastic Programming	Metaheuristic algorithm-Weighted sum method
This article	✔	✔		✔		✔	Stochastic Programming	Metaheuristic algorithm-Ideal-point-method

In summary, this paper fully considered carbon emission sources, including transportation carbon emission from the perspective of real-time fuel consumption, fixed carbon emission of logistics centers, and variable carbon emission of logistics centers, established a multi-objective planning mathematical model of logistics cost and carbon emission, and designed an AFO-ideal point algorithm to solve the multi- objective model. Through case analysis, it is demonstrated that the proposed optimization algorithm can effectively solve the logistic center location problem.

## 3.Problem definition and mathematical formulation

In this section, we construct a model for the location of low-carbon logistics centers considering demand uncertainty. In Section 3.1, we introduce the model framework for the low-carbon logistics center siting problem in the context of demand uncertainty. Subsequently, in Section 3.2, we construct the location model in further detail based on the relationship between fuel consumption and carbon emissions.

### 3.1. Problem description

**Fig 1:**
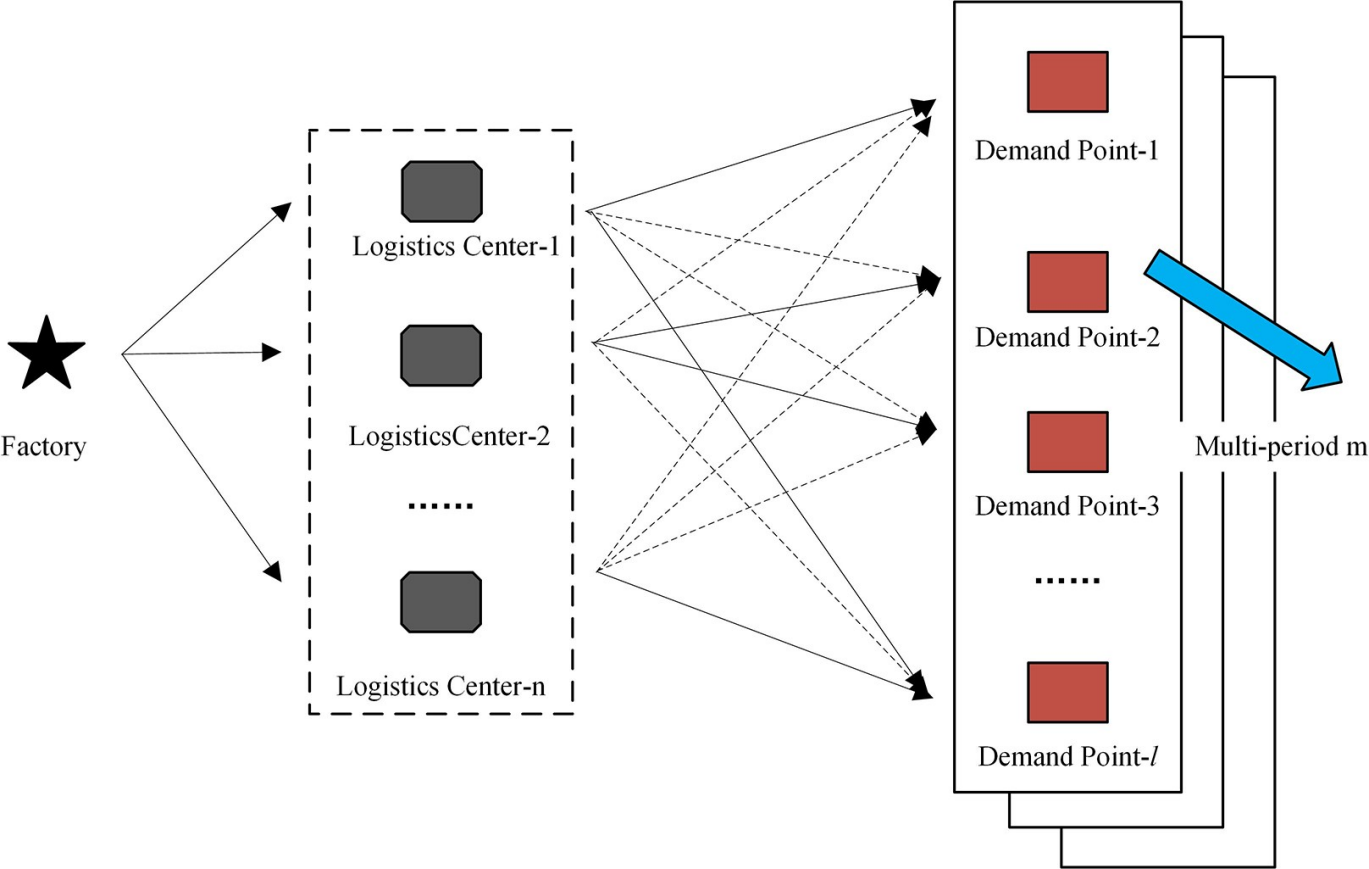
Multi-period single-source three-level logistics and distribution network.

Consider the factory, alternative logistics center, and demand point to establish a single commodity multi-period single-source three-level logistics and distribution network model, as shown in Fig 1. The goods during the period are distributed to different demand points after flowing through the logistics center. The demand for each demand point is uncertain, the factory is well stocked, and the distance and freight costs between the nodes are known. Considering the fixed cost of alternative logistics center construction, cargo transportation cost and carbon emission, we establish the multi-objective mathematical model of logistics cost and carbon emission. The symbols used in this paper are shown in Table 3. To facilitate the research calculations, the following assumptions were made:

**A1.** Assuming that the mode of transport at each stage of the transport process is a single mode of transport [37].
**A2.** The cost of transportation is proportional to the weight of the goods and the distance between the nodes [12].
**A3.** The demand for goods at each demand point is independent of each other and follows a normal distribution [5–15].
**A4.** The construction of each alternative logistics center, considering only the expansion costs and the distribution center’s variable carbon emissions related to a concave function of cargo flow [37].
**A5.** To simplify the calculation, we assume the same cargo handling capacity as the alternative logistics center, with no limitation on cargo transportation capacity.
**A6.** The vehicle specifications are the same [38], and the vehicle travels at a uniform speed during transportation [39].

**Table 3:** Parameter description.

Notations	Description
Sets	
*J*	Set of alternative logistics centers *j*, *j*∈*J*
*K*	Set of demand points *k*, *k*∈*K*
Parameters	
*q*_*k*_	Amount of cargo shipped to demand point *k* during the cycle, unit: t.
*S*_*j*_	Distance between factory and logistics center *j*, unit: km
*S*_*jk*_	Distance from logistics center *j* to demand point *k*,unit: km
*c*_*j*_	Freight rates from factory to logistics center *j*,unit: ¥/(*km*∙*t*)
*c*_*jk*_	Freight rates from logistics centers *j* to demand point *k*,unit: ¥/(*km*∙*t*)
*μ*_*j*_	*μ*_*j*_ is the flow rate of alternative logistics center *j*, unit: t
*γ*	Fuel carbon emission factors, (kg·CO_2_/kg)
*H*_*j*_	Logistics center changes in carbon emissions, (kg·CO_2_/t).
*m*	Demand cycle for goods, where m is a constant.
*Q*_*j*_	Capacity ceiling for logistics center *j*, unit: t.
Uncertain parameters	
*μ*_*k*_	Demand for commodities at demand point k in a single cycle.
Decision Variables	
$Z_{j}\left\{ \begin{array}{c} 1\\ 0 \end{array}\right.$	Alternative logistics center j was selected if *Z*_*j*_ = 1, and 0 otherwise.
$X_{j}^{k}\left\{ \begin{array}{c} 1\\ 0 \end{array}\right.$	Logistics center *j* delivers to demand point *k* if $X_{j}^{k}$ = 1, and 0 otherwise.

Remark: The letter t is an abbreviation for tons.

### 3.2 Mathematical formulation

This paper constructs a multi-objective mathematical model of low-carbon logistics center location with logistics cost *C* (transportation cost *C*_1_, logistics center fixed construction cost *C*_2_) and carbon emission *E*.

#### 3.2.1 Logistics costs

The logistics cost in a single period is composed of transportation cost C1, site selection fixed cost ***C***_**2**_, which is expressed by Eqs (1) and (2), so the period logistics cost C can be expressed by Eq (3).


$$C_{1}=\sum_{j\in J}\sum_{k\in K}S_{j}c_{j}q_{k}X_{j}^{k}Z_{j}+\sum_{j\in J}\sum_{k\in K}S_{jk}c_{jk}q_{k}X_{j}^{k}Z_{j}$$ (1)

$$C_{2}=\sum_{j\in J}Z_{j}C_{j}$$ (2)

$$\min C=mC_{1}+C_{2}$$ (3)

#### 3.2.2 Carbon emissions

The logistics location process generates significant carbon emissions, mainly from the transportation of goods and from the operation of logistics centers. Carbon emissions from the transportation process are mainly from engine fuel consumption. In this paper, combining the fuel consumption calculation method of Xiao [40] and the instant fuel consumption models of Ben-Chaim [41] and Bowyer [38], it can be obtained that the instant fuel consumption ***f***_***L***_ can be calculated by Eqs (4) and (5) shown.

$$f_{L}=\frac{f_{\max}-f_{0}}{M_{\max}}q_{k}+f_{0}$$ (4)

$$f=\rho \beta_{1}b_{1}+\rho \beta_{1}b_{2}v^{2}+\frac{\rho \partial }{v}+\frac{9.81\rho \beta_{1}MG}{100000}$$ (5)

*f*_*max*_, *f*_0_ are the fuel consumptions of fully loaded and unloaded vehicles, respectively. *M*_*max*_ is the maximum load weight of the vehicle. *m* indicates the actual weight of the vehicle. *ρ* is the density of the fuel used in the vehicle. *β*_1_ is the engine energy efficiency factor. *b*_1_ is the resistance of rolling friction. *b*_2_ indicates rolling pneumatic force. *M* indicates vehicle weight. *G* denotes slope. ∂ denotes fuel idle consumption rate.

Therefore, the transport carbon emission *E*_1_ can be expressed by Eq (6).


$$\begin{array}{l} E_{1}=\gamma \sum_{j\in J}\sum_{k\in K}S_{j}(\frac{f_{\max}-f_{0}}{M_{\max}}q_{k}+f_{0})X_{j}^{k}Z_{j}+\\ \gamma \sum_{j\in J}\sum_{k\in K}S_{jk}(\frac{f_{\max}-f_{0}}{M_{\max}}q_{k}+f_{0})X_{j}^{k}Z_{j} \end{array}$$ (6)

Carbon emissions from distribution centers can be divided into fixed carbon emissions *E*_2_ and variable carbon emissions *E*_3_. Fixed carbon emission refers to the carbon emissions of lighting, constant temperature, and mechanical operation in distribution centers, where lighting and constant temperature are dominated by electrical energy consumption while mechanical operation is based on two sources of electrical energy and fuel consumption. Variable carbon emissions are related to the amount of goods handled.

In this paper, we refer to the carbon emission calculation model proposed by the Intergovernmental Panel on Climate Change (IPCC) to obtain the fixed carbon emissions of distribution centers as shown in Eq (7). From the discussion in Sections 2.1 and 2.2.2, the carbon emissions of distribution center changes are shown in Eq (8).

$$E_{2}=e_{w}\sigma_{1}+\frac{44}{12}e_{j}\sigma_{2}\sigma_{3}\sigma_{4}$$ (7)

$$E_{3}=\sum_{j\in J}{\left(\mu_{j}\right)}^{\theta }Z_{j}H_{j}$$ (8)

where *e*_*w*_ is the electrical energy consumed by the logistics center *e*_*j*_ is the amount of fuel consumed by the logistics center. *σ*_1_ is the logistics center carbon conversion constant. *σ*_2_ is the low-level heating value per unit of fuel. *σ*_3_ is the carbon content per unit calorific value of fuel. *σ*_4_ is the carbon oxidation rate of fuel (a constant between 0 and 1). *H*_*j*_ is the carbon emission per unit weight of cargo handled by the forklift in the logistics center. *θ* is a constant, *θϵ*(0,1).

Therefore, the carbon emission E of the logistics location in the period can be expressed by Eq (9).


$$E=m(E_{1}+E_{2}+E_{3})$$ (9)

#### 3.2.3 Model construction


$$\{\begin{array}{c} \min C=mC_{1}+C_{2}\\ \min E=m(E_{1}+E_{2}+E_{3}) \end{array}$$ (10)

Subject to:

$$\sum_{j\in J}\sum_{k\in K}q_{k}X_{j}^{k}Z_{j}=\sum_{k\in K}q_{k}$$ (11)

$$\sum_{k\in K}q_{k}\leq \sum_{j\in J}Z_{j}Q_{j}$$ (12)

$$\sum_{j\in J}X_{j}^{k}=1$$ (13)

$$P_{r}\{\mu_{k}\leq q_{k}\}\geq \alpha$$ (14)

$$\mu_{j}=\sum_{j\in J}\sum_{k\in K}q_{k}X_{j}^{k}Z_{j}$$ (15)

$$X_{j}^{k}\leq Z_{j}$$ (16)

$$q_{k}\geq 0$$ (17)

The objective function (10) indicates that logistics costs and carbon emissions are minimized. Constraint (11) states that the flow balance in and out of the logistics center. Constraint (12) states the logistics center’s cargo handling capacity constraint. Constraint (13) stated that each demand point will be satisfied. Constraint (14) states that in the case of demand uncertainty, it is not required to satisfy all demands by meeting all scenarios, but as long as the probability of satisfying all demands is greater than *α*. Constraint. (15) represents the cargo throughput of the logistics center. Constraint (16) stated that the alternative logistics center *j* can distribute cargo to demand point *k* only if it is selected. Constraint (17) states that cargo volume should be positive.

#### 3.2.4 Stochastic constraint transformation

In performing the equivalent transformation of stochastic programming, this paper refers to the study of scholars in Table 1 and assumes that the demand probability distribution of demand points obeys $N(\mu_{k},\sigma_{k}^{2})$. We convert the multi-objective mathematical model into a single-objective mathematical model utilizing the ideal point method. First, the model is solved with the objective of minimizing the logistics cost ***y***_**1**_, and the optimal solution $y_{1}^{*}$ is obtained. Then the model is solved with the objective of minimizing transport carbon emissions ***y***_**2**_ to obtain the optimal solution $y_{2}^{*}$. We construct the new optimization objective $y=\sqrt{{\left(y_{1}-y_{1}^{*}\right)}^{2}+{\left(y_{2}-y_{2}^{*}\right)}^{2}}$ by combining the ideal values of the above objectives, and transform the original multi-objective optimization problem into a single-objective optimization problem. Finally, with ***y*** as the new objective, the solution is again combined with the model constraints. Only the demand parameter ***q***_***k***_ is an uncertain parameter in the model for each solution. We assume that the demand point satisfies the full demand probability ***α*** is sufficient. And the constraint (14) constraint is difficult to solve. We refer to Yang [42] for an equivalent conversion method for stochastic programming, we convert the stochastic constraint (14) into a chance constraint. If we make *T* = *μ*_*k*_−*q*_*k*_, then the expectation and variance of T are represented by Eqs (18) and (19), respectively.


$$E(T)=E(\mu_{k})-q_{k}$$ (18)

$$D(T)=D(\mu_{k})$$ (19)

If we make $\psi =\frac{T-E(T)}{\sqrt{D(T)}}$, then *ψ*~*N*(0,1).Then *T* = *μ*_*k*_−*q*_*k*_ is equivalent to $\psi =\frac{T-E(T)}{\sqrt{D(T)}}\leq -\frac{E(T)}{\sqrt{D(T)}}$. Then the constraint (14) can be expressed in terms of $P_{r}\{\psi \leq -\frac{E(T)}{\sqrt{D(T)}}\}\geq \alpha$.

We assume that the probability density function of *ψ* is Φ(*ψ*). If constraint (14) holds at confidence level *α*, it follows from Eqs (18) and (19) that constraint (14) can be transformed into constraint (20) when and only when a = b. If constraint (14) holds at confidence level a, from constraints (18) and (19), when and only when $\Phi {\left(\psi \right)}^{-1}⩽-\frac{E(T)}{\sqrt{D(T)}}$ holds, then constraint (14) can be transformed into constraint (20).


$$\Phi {\left(\psi \right)}^{-1}\sqrt{D(T)}+E(T)\leq q_{k}$$ (20)

#### 3.2.5 Distance calculation

To improve the accuracy of the distance, we use the spherical distance formula to solve for the distance between nodes. We let the latitude and longitude of two nodes A and B be (***ϕ***_**1**_, ***φ***_**1**_) and (***ϕ***_**2**_, ***φ***_**2**_), respectively, and then the distance d between the two points A and B is shown in Eq (21).

$$d=R\arccos[\cos\phi_{1}\cos\phi_{2}\cos(\varphi_{1}-\varphi_{2})+\sin\phi_{1}\sin\phi_{2}]$$ (21)

where R is the spherical radius, and in this paper R = 6371km.

## 4. Solution methodology

In this section, we combine the AFO algorithm proposed by Yang [22] with the ideal point method to design algorithms for solving the multi-objective planning problem of siting logistics centers with uncertain demand. We refer to Fathollahi-Fard [43] and Tian [44] to describe our algorithm in terms of the sequence of generating the initial solution, the optimization method, and constraint verification. In Section 4.1, we introduce in detail the solution representation of the problem solved by the algorithm as well as the optimization strategy. In Section 4.2, it is explained in detail how the constraints on the algorithm as well as the model can be restricted. Finally, in Section 4.3, we illustrate how the AFO algorithm can be combined with the ideal point method to solve the multi-objective problem of low-carbon logistics center location under demand uncertainty.

### 4.1 AFO algorithm

#### 4.1.1 Solution representation and search space optimization

For most metaheuristics, the algorithms are designed for continuous-type problems and cannot solve discrete-type problems directly. The logistics site selection problem in this paper is a combinatorial optimization problem, i.e., a discrete problem. To solve the discrete problem using a continuous metaheuristic algorithm, we define penguins as representatives of the solution to a given optimization problem, i.e., each penguin represents a set of logistics location options. Specifically, real number encoding is performed, with an encoding length of *j*+*k* (where j is the number of alternative logistics centers and k is the number of demand points).

The AFO algorithm utilizes a gradient estimation strategy in the optimization search process. Specifically, the penguins are influenced by five factors in the process of finding the optimal temperature: their own temperature sensing, their own memory influence, the influence of their own peers, approaching the penguin center, and keeping their energy loss to a minimum, which can be divided into three strategies due to different objects. Influencing factor Ⅰ is adopted at a certain interval at the center of a separate penguin population, so movement strategy Ⅰ is mainly used in the search for superiority at the center of the population; penguins at the center of the penguin population have a higher probability of being influenced by their own kind to move, so factor Ⅲ is mainly adopted by penguins at the center of the penguin population; the remaining movement patterns Ⅱ, Ⅳ and Ⅴ are grouped together, so the five influencing factors can be divided into three movement strategies $X_{ci}^{t},X_{i2}^{t},X_{i3}^{t}$.

The movement strategy Ⅰ is dedicated to the displacement update iteration at the center of the penguin population, and the displacement change of movement strategy Ⅰ is shown in Eq (22).

$$AT_{i1}^{t}=\alpha_{i}{\tilde{G}}_{i}^{'t}$$ (22)

where ${AT}_{i1}^{t}$ indicates the displacement change influenced by its own temperature-sensitive factors; *α*_*i*_ is the conversion factor from gradient to displacement; and ${\tilde{G}}_{i}^{'t}$ is the gradient change of Penguin i at moment t.

The correlation parameters are calculated as in Eqs 23–30, N is the penguin population size, D is the decision variable dimension *S*_*max*_, *S*_*min*_ denote the maximum and minimum values of the displacement of penguin j, respectively; Δx is the increment of displacement change; *c* is the step change coefficient of gradient estimation.

At moment t, the directional displacement change of penguin i along direction j is positive and negative for $X_{ij}^{'t}$ and $X_{ij}^{"t}$, respectively, and the corresponding temperature changes are $Y_{ij}^{'t}$ and $Y_{ij}^{"t}$.


$$X_{ij}^{'t}=[x_{1},\ldots ,x_{j}+\Delta x,\ldots x_{D}]$$ (23)

$$X_{ij}^{"t}=[x_{1},\ldots ,x_{j}-\Delta x,\ldots ,x_{D}]$$ (24)

$$\Delta x=\frac{c\|S_{\max}-S_{\min}\|}{2}$$ (25)

$$c=\exp(-\frac{30(t-gap_{0})}{M}$$ (26)

$${\tilde{g}}_{ij}^{t}=\frac{\log Y_{ij}^{'t}-\log X_{ij}^{"t}}{\Delta x}$$ (27)

$${\tilde{G}}_{i}^{t}=[{\tilde{g}}_{i1}^{t},\ldots ,{\tilde{g}}_{ij}^{t},\ldots ,{\tilde{g}}_{iD}^{t}]$$ (28)

$$\alpha_{i}=\exp(-\frac{10(i-1)}{N-2D-1})(1-\frac{\left(i-1)(1-\frac{\left|\Delta x\right|}{\left\|{\tilde{G}}_{i}^{'t}\right\|}\right)}{N-2D-1})$$ (29)

$${\tilde{G}}_{i}^{'t}=\frac{{\tilde{G}}_{i}^{t}\|S_{\max}-S_{\min}\|}{2\|{\tilde{G}}_{i}^{t}\|}$$ (30)

Considering the actual situation, the objective function of some optimization problems is not differentiable, or in some special cases, the gradient is estimated to be 0. At this time, the original displacement update formula can no longer be used, and at this time Eq (22) can be replaced by $AT_{i1}^{t}=\frac{5D_{m}R_{n}}{\left\|R_{n}\right\|}$, where *D*_*m*_ is the average distance from all penguins in the population to the penguin center and *R*_*n*_ is a random matrix obeying normality (dimensionality is consistent with $X_{i}^{t}$).

Therefore, the displacement change in the case of strategy Ⅰ can be expressed as $X_{ci}^{t}=X_{ci}^{t-1}+AT_{i1}^{t}$.

Movement strategy II: Penguins at the center of the penguin population have a higher probability of moving under the influence of their own kind, so strategy II is mainly adopted by penguins at the center of the penguin population, and the newly generated position will replace the position of each penguin in the population. The change in displacement of a penguin under the influence of its own kind is related to the standard deviation *std*_*j*_ of the jth penguin’s memory position, which is updated using Eq (31) if $std_{j}\leq \exp(-\frac{20t}{M})(S_{\max}-S_{\min})$, Eq (32); if $Ym_{p1}^{t}=Ym_{p2}^{t}$, and Eq (33) otherwise.

$$AI_{i3}^{t}=-0.5R_{2}\lambda (S_{\max j}-S_{\min j})(Xm_{p1j}^{t}-Xm_{p2j}^{t})(-\mathrm{sgn}(Ym_{p1}^{t}-Ym_{p2}^{t}))$$ (31)

$$AI_{i3}^{t}=\frac{R_{n}\lambda (S_{\max j}-S_{\min j})}{2}$$ (32)

$$AI_{i3}^{t}=R_{2}(Xm_{p1j}^{t}-Xm_{p2j}^{t})(-\mathrm{sgn}(Ym_{p1}^{t}-Ym_{p2}^{t}))$$ (33)

where ${AI}_{i3}^{t}$ denotes the change in displacement under the influence of other individual factors. *R*_2_ is a random matrix obeying a 0–1 uniform distribution (the dimensions are consistent with $X_{i}^{t}$). ${Xm}_{p1j}^{t}$ and ${Xm}_{p2j}^{t}$ denote the best positions in the memory of two randomly selected penguins at time t, respectively, and the corresponding best temperatures are ${Ym}_{p1}^{t}$ and ${Ym}_{p2}^{t}$. *S*_*maxj*_、and *S*_*minj*_ state the maximum and minimum values of displacement movement of penguin j, respectively. *sgn*(*X*) is a symbolic function that determines the positive and negative of a variable and makes the positive and negative values consistent with X; $\lambda =2\arctan h(1-\frac{t}{M})$; Therefore, the displacement change under Moving strategy II is $X_{i2}^{t}=X_{ci}^{t-1}+AI_{i3}^{t}$.

Movement strategy Ⅲ: Strategy Ⅲ is formed by the action of three factors, movement patterns Ⅱ, Ⅳ and Ⅴ, and applies to all individuals in the population, The displacement can be expressed as $X_{i3}^{t}=X_{i}^{t-1}+\omega_{2}AM_{i2}^{t}+\omega_{4}AC_{i4}^{t}+\omega_{5}AV_{i5}^{t}$.


$$AM_{i2}^{t}=R_{1}\beta (Xm_{i}^{t}-X_{i}^{t})$$ (34)

$$AC_{i4}^{t}=R_{3}\delta (X_{c}^{t}-X_{i}^{t})$$ (35)

$$AV_{i5}^{t}=\varepsilon A_{i}^{t-1}$$ (36)

Eqs (25)–(30) represent the update formulas for moving models II, IV, and V. Where ${AM}_{i2}^{t}$ denotes the change in displacement influenced by its own memory factors. *R*_1_ is a random matrix obeying a 0–1 uniform distribution (the dimensions are consistent with $X_{i}^{t}$). ${Xm}_{i}^{t}$ is the best position of temperature in penguin memory. *β* is the memory influence coefficient.; ${AC}_{i4}^{t}$ indicates the change in displacement of the penguin influenced by the factor of approaching the center.*R*_3_ is a random matrix obeying a 0–1 uniform distribution (the dimensions are consistent with $X_{i}^{t}$); *δ* is the coefficient of influence of penguin population centre; X is the position of the penguin centre; where ${AV}_{i5}^{t}$ denotes the displacement change that keeps its own energy loss minimized. *ε* is the last displacement influence factor.

In practice, the interval gap of the penguin individual using strategy I will gradually decrease with the increase in the number of iterations. The initial time interval for updating the displacement change using strategy I is a, where a is related to the maximum number of iterations of the algorithm, as shown in Eq (37). Gap is iterated as shown in Eq (38), and if the number of iterations t is an integer multiple of gap, then strategy I is used for displacement update.

$$gap_{0}=ceil(\sqrt{M})+1$$ (37)

$$gap_{t}=\max(gap_{\min},gap_{t-1}-dec)$$ (38)

where *ceil* is the upward rounding function. *dec* (*dec* is a constant) is the decreasing value of the interval time of strategy I. *gap*_*min*_ is the minimum trigger time interval of strategy I updates (*gap*_*min*_ is a constant).

The probability of adopting strategy III for the i-th penguin in the population in iteration period t is *p*_*i*_; *p*_*i*_ is calculated as in Eq (39), and the probability of adopting strategy II is *p* = 1−*p*_*i*_.


$$p_{i}=\arctan h(|Y_{i}^{t}-Y_{c}^{t}|)\frac{M-t}{M}$$ (39)

#### 4.1.2 Strategies to prevent premature convergence

In the current iteration period, if the temperature at the center of the penguin population changes less than 5% from the previous period, the current iteration period is considered to have prematurely converged and a local optimum has occurred. When the number of premature convergences reaches a certain threshold L, the algorithm performs an anti-premature convergence strategy. We regenerated the positions of all penguins except one at the center of the population and cleared their memories. This cumulative process is expressed in mathematical equations as Eqs (40) and (41). If ***count***≥***L*** (where L is a constant), a premature convergence prevention strategy will be carried out.


$$0.95X_{c}^{t-1}\leq X_{c}^{t}$$ (40)

count=count+1 (41)

From sections 4.1.1 and 4.1.2, we can obtain the optimal search process of the algorithm. In the actual case, if the temperature $Y_{i}^{t}>Y_{i}^{t-1}$ before and after the movement is the same, then it is considered that the current displacement of the penguin has generated an error and produced a solution that does not meet the requirements, and at this time, we use Eq 33 to regenerate the displacement change and continue to search for the optimal solution. When the maximum number of iterations is reached, the penguin population center position and the corresponding optimal solution are determined.

From the descriptions in Sections 4.1.1 and 4.1.2, the AFO algorithm pseudocode can be found in S1 Appendix.

### 4.2 Constraint verification

The algorithm proposed in this paper for constraint validation mainly consists of two parts: one part is based on the decoding method based on real number priority adopted in this paper, and the specific decoding process is as follows for each individual *Xi* in the population N that satisfies the maximum number of iterations:

**Table:** 

Step1: For individual *X*_*i*_, logistics center decoding: for the logistics center, for the real number of the code in ascending order, according to the number of logistics centers determined by the algorithm b, select the smallest real value of the first b number as the corresponding determined logistics center.
Step2: Distribution scheme decoding: In this paper, the distribution scheme is determined by prioritizing the distance *S*_*jk*_ between nodes. The real values are sorted in ascending order after encoding, and the bit order represents the distribution order.
Step3: Compare the distance *S*_*jk*_ between demand point *k* and logistics center *j* and determine the distribution center for distribution according to the distance priority (the smaller the *S*_*jk*_, the higher the priority).
Step4: Calculate the remaining capacity of the distribution center *Load* = *Q*_*j*_−*q*_*k*_ (the initial value of *Q*_*j*_ is the upper limit of the logistics center capacity).
Step5: While *k*≤*l*, go to step 2, otherwise go to step 6.
Step6 If Load>0 and all demands are distributed, this distribution scheme is feasible; if there are unmet demands from distribution centers and *Load*≤0, all unmet demands *Pn* are recorded.
Step7: Calculate the fitness function at this point according to Eq (42). If *i*>*N*, then go to Step8, otherwise go to Step2
Step8: Output the best individual *X*_*best*_ in the population and the distribution scheme after the above decoding scheme.

The coding approach used in this paper transfers the model constraints to the decoding layer, which enables the satisfaction of constraints (11), (12), (15), (16), and (17). For the remaining constraints (13) and (20), we realize them through penalty functions. Specifically, we ensure that all demand constraints are satisfied by calculating the total number of unsatisfied distribution demands at the demand point, *P*_*n*_, and then assigning *P*_*n*_ a larger coefficient *τ*, which is then added to the fitness function, at which point unsatisfied solutions are penalized with a larger penalty.

### 4.3 AFO—Ideal point method

The ideal point method is a method for solving multi-objective optimization problems. The basic principle is to find a special point called the "ideal point", which is the minimum vector of all objective function values (i.e., the optimal solution of all objective function values), and then transform the problem into a process of finding a set of optimal solutions that are closest to the ideal point.

**Fig 2:**
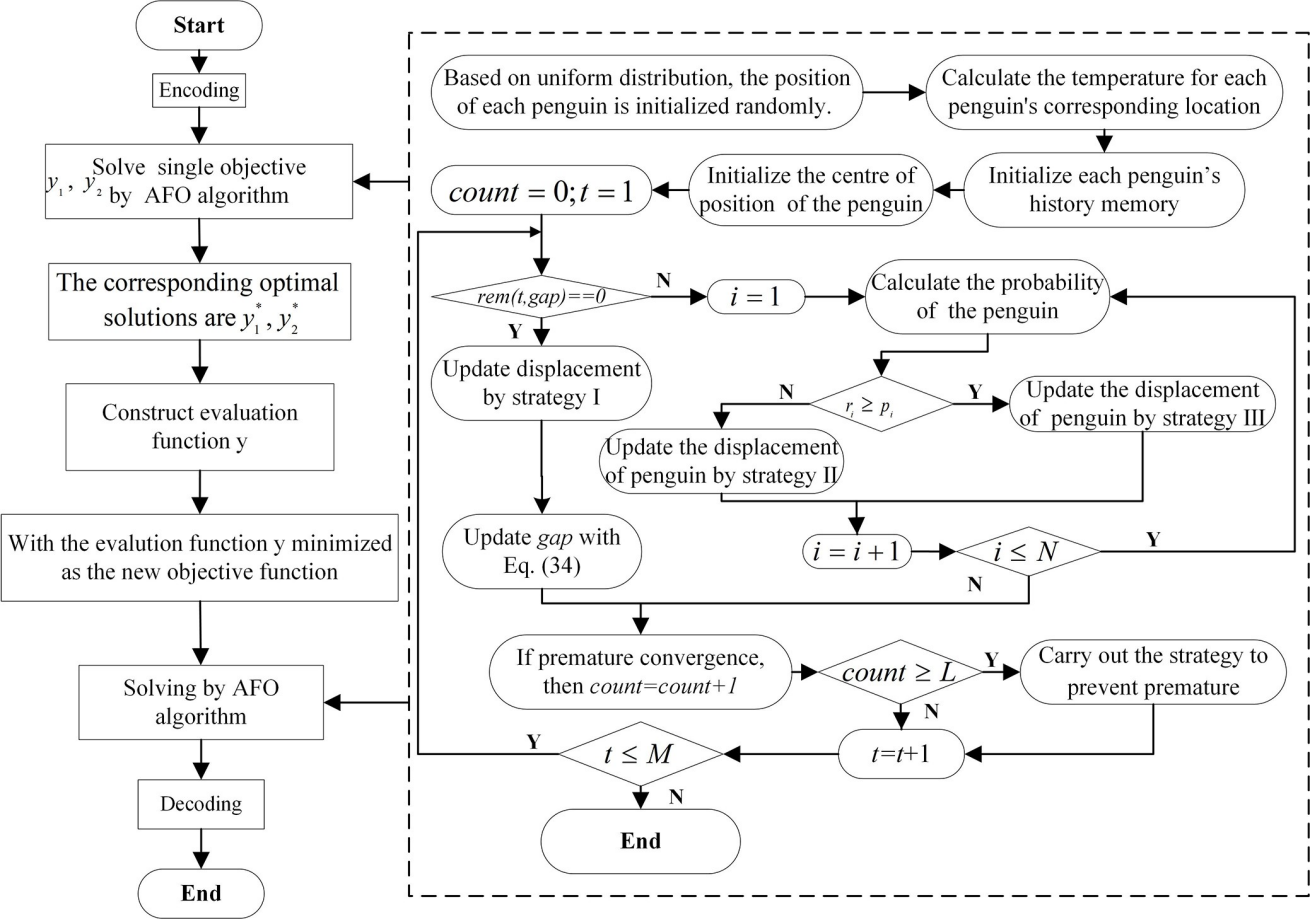
AFO-ideal point method algorithm flowchart.

Synthesizing what is described in Section 3.2.4, Section 4.1, and Section 4.2, the flowchart of the AFO-ideal point method is shown in Fig 2. This paper the algorithmic fitness function is expressed as an evaluation function and can be represented by Eq (42).


$$y=\sqrt{{\left(y_{1}-y_{1}^{*}\right)}^{2}+{\left(y_{2}-y_{2}^{*}\right)}^{2}}+\tau p_{n}$$ (42)

In addition, the comparison algorithm proposed below as well as the exact solver (LINGO) are combined with the ideal point method to construct the evaluation function shown in Eq (42), which is then solved by the algorithm.

## 5. Case study

In this section, we describe in detail how the AFO-ideal point method can be applied to a real-life case of supermarket retail logistics site selection. In Section 5.1, we describe the case data and the relevant parameter settings; in Section 5.2, we apply the AFO algorithm to solve the case and compare the results with four other classical heuristics; and in Section 5.3, we perform a sensitivity analysis of the freight costs and their uncertain parameters.

### 5.1 Data description

**Fig 3:**
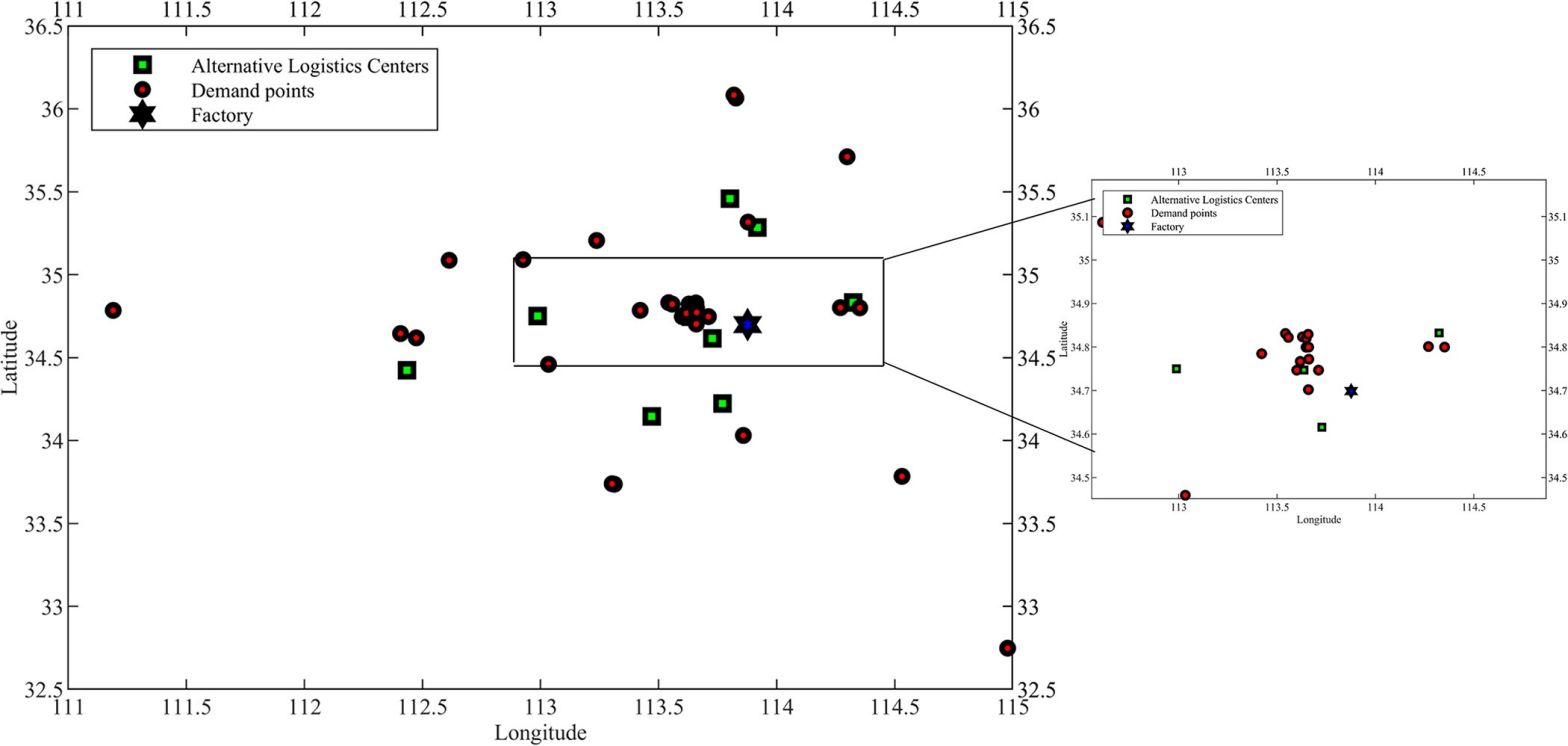
Distribution of nodes in the area.

We take supermarket retail logistics location distribution as an example from the data set of cargo demand reference Maoni [37].Taking the monthly demand of 42 stores in the region as the period, the demand points are numbered as 1, 2, 3…42 in order, and the logistics centers are also demand points according to the actual situation, and the demand points 12, 17, 24, 26, 29, 35, 38, 39, and 42 are numbered as alternative logistics centers in order as P1, P2,…P9, S is the supply point, and the upper limit of capacity of each logistics center is 59 tons (We set the upper limit of the capacity of each logistics center to be the same, and the capacity of the logistics center is the average of the capacity of the data set.). The location scheme is studied over a one-year period, i.e., m = 12. The logistic center and vehicle-related constants are shown in Table 4, and the alternative logistic center information is shown in Table 5. Through the discussion in Section 1 and Section 2.4, it is assumed that the demand for cargo at the demand point obeys a normal distribution $N(\mu_{k},\sigma_{k}^{2})$., and the basic information of the demand point and the demand distribution are shown in S1 File. The distribution of each node is shown in Fig 3. Assume that the vehicle travels at a uniform speed of 40 km/h.

**Table 4:** Logistics center and transport vehicle related parameters.

Distribution center related parameters		Vehicle related parameters	
*σ*_1_	0.9515(t·C/MWh)	*ρ*	0.085(kg/km)
*σ*_2_	42652(KJ/kg)	*β*_1_	0.08(ml/KJ)
*σ*_3_	20.2(t·C/TJ)	*b*_1_	0.698(kN)
*σ*_4_	0.98	*b*_2_	0.00358(kN/m/s^2^)
*γ*	3.1(kg·CO_2_/kg)	∂	0.527(ml/s)
*θ*	0.5	*G*	0.1
*H*_*j*_	0.2(kg/t)	*M*_*max*_	25000(kg)
-	-	*m*_0_	8000(kg)

Remark: *σ*_1_ refers to the"2015 China Regional Grid Baseline Emission Factor"; *H*_*j*_ is the carbon emission per unit of cargo handled by fuel forklift in the logistics center. *σ*_2_, *σ*_3_, *σ*_4_, *γ* data sources "General Rules for Calculating Comprehensive Energy Consumption" (GB/T 2589–2008) and "Guidelines for Preparing Provincial Greenhouse Gas Inventories" (NDRC Climate [2011] No. 1041. The vehicle-related parameters are obtained from the relevant studies of Xiao [40], Ben-Chaim [41] and Bowyer [38]. The value of *θ* is taken from "The Difference and Application of Variable Cost Method and Full Cost Method".

**Table 5:** Alternative logistics center basic information.

Alternative Logistics Center	Longitude	Latitude	Fixed cost/¥ (10^4^)	*e*_*w*_ (KWh)	*e*_*j*_ (kg)
P1	113.727989	34.615547	84.93	1420	12
P2	112.98816	34.75003	86.41	1340	14
P3	113.770287	34.223457	56.69	1033	11
P4	114.323753	34.832211	74.76	1833	9
P5	112.434761	34.42276	56.85	1032	18
P6	113.470589	34.145721	56.9	1462	16
P7	113.91864	35.283562	75.58	1051	17
P8	113.802055	35.4576	56.7	1320	13
P9	113.636429	34.74715	135.45	1630	16
S	113.876892	34.698662	--	--	--

Remark: *e*_*w*_ from http://www.chinapower.com.cn/; *e*_*j*_ refer to the fuel consumption of machinery and equipment in the logistics center.

Referring to the transport rate standard of domestic EMS, we set the transport rate, *c*_*j*_ = *c*_*jk*_ = 10(¥/km·t)), set the number of populations N = 30, the maximum number of iterations M = 200, ω_2_ = 0.5, ω_4_ = 1, ω_5_ = 1, L = 15, *gap*_*min*_ = 5, *dec* = 2; Referring to the transport rate standard of domestic EMS, we set the transport rate, *c*_*j*_ = *c*_*jk*_ = 10(¥/km·t)), set the number of populations N = 30, the maximum number of iterations M = 200, ω_2_ = 0.5, ω_4_ = 1, ω_5_ = 1, L = 15, *gap*_*min*_ = 5, *dec* = 2. For the PSO algorithm, set the parameter inertia weight ω to 0.65, the cognitive learning factor *c*_1_ to 0.65, and the social learning factor *c*_2_ to 0.65. For the DE algorithm, set the scaling factor F to 0.4 and the crossover probability CR to 0.5. For the TS algorithm, set the algorithm tabu list to 100. For the SS algorithm, set the discoverer percentage (PD) to 0.2, the scout percentage (SD) to 0.1, and the switch probability (ST) to 0.8, and run based on matlab2022b.

### 5.2 Analysis of case results

#### 5.2.1 Location scheme under demand determination situation

**Fig 4:**
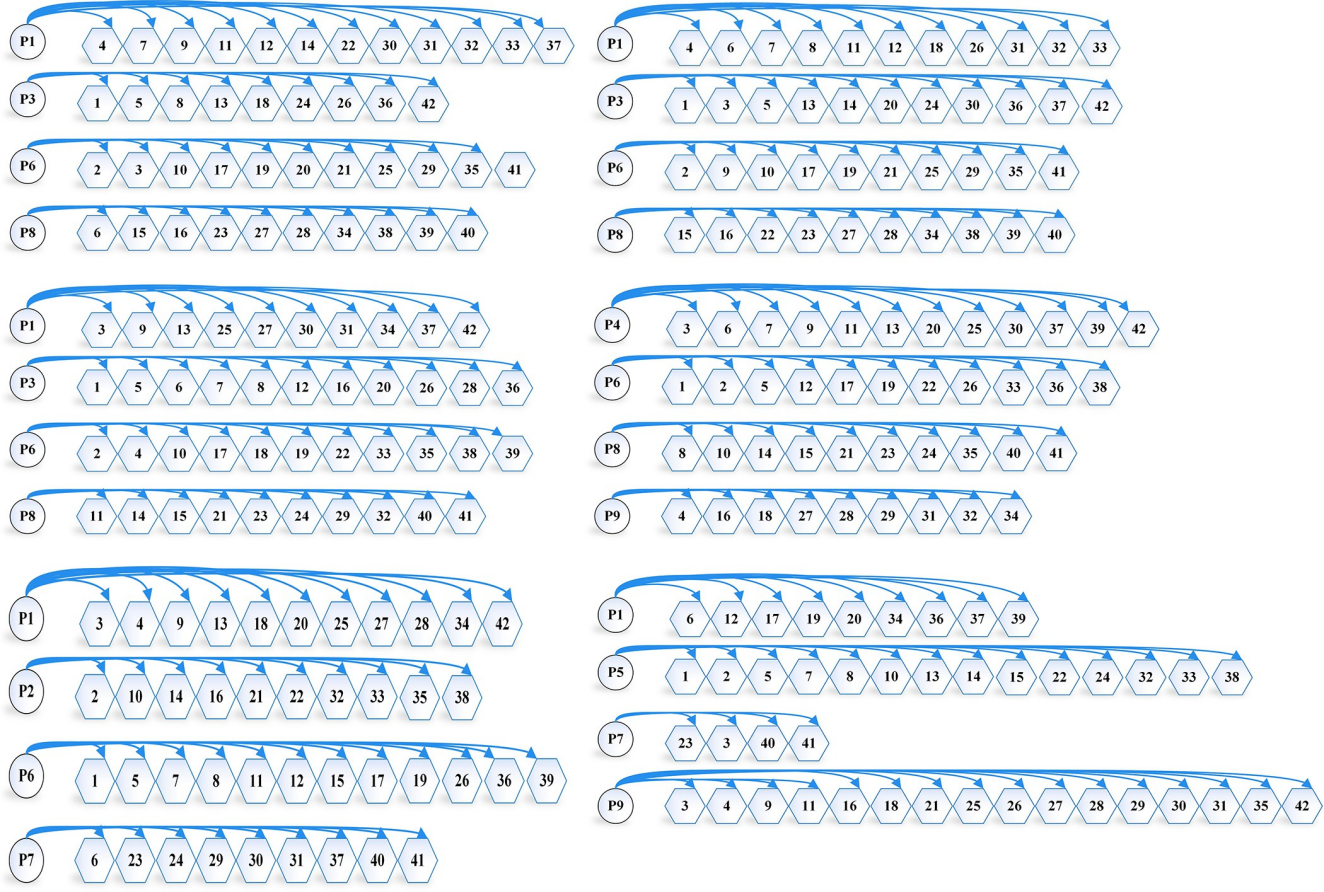
Distribution schemes under demand determination. (a) Distribution scheme determined by AFO algorithm. (b) Distribution scheme determined by PSO algorithm. (c) Distribution scheme determined by DE algorithm. (d) Distribution scheme determined by TS algorithm. (e) Distribution scheme determined by SS algorithm. (f) Distribution scheme determined by LINGO.

Firstly, we study the location scheme under demand determination (using the average value of demand as cargo demand) and solve the model using the AFO algorithm, with the objective of minimizing logistics cost ***y***_**1**_, and obtain $y_{1}^{*}$ = 566.42(¥·10^4^). We then solve the model with the objective of minimizing transportation carbon emission ***y***_**2**_, and obtain $y_{2}^{*}$ = 80.72(t). At this situation ***P***_***n***_ = **0**, the evaluation function $y=\sqrt{{\left(y_{1}-566.42\right)}^{2}+{\left(y_{2}-80.72\right)}^{2}}$. The original multi-objective optimization problem is transformed into a single-objective optimization problem, which is again solved by combining the model constraints. Similarly, PSO, DE, TS, SS algorithms, and LINGO are used to solve the single-objective solution first and then construct the evaluation function to convert the multi-objective function into a single-objective optimization model and then solve it again. The distribution scheme solved by the six methods is shown in Fig 4, the comparison of the solution results of the the six methods is shown in Table 6, and the algorithm iteration diagram as shown in Fig 6.

**Table 6:** Comparison of algorithm solution under demand determination.

Algorithm	Fixed cost (¥·10^4^)	Transport cost (¥·10^4^)	Logistic cost (¥·10^4^)	Carbon emissions (t)	Computational time(s)
AFO	255.22	311.06	566.28	84.02	4.9
PSO	255.22	311.75	566.97	84.23	2.5
DE	255.22	312.59	567.81	84.77	2.7
TS	323.81	314.68	638.49	96.29	4.0
SS	303.82	303.44	607.26	85.16	2.6
LINGO	352.81	258.18	637.99	85.14	17

From Fig 4 and Table 6, we can see that the AFO algorithm, PSO algorithm, and DE algorithm determine the same number and location of logistics centers under demand determination, but the distribution scheme is different. The AFO algorithm outperforms the PSO algorithm and the DE algorithm in the decision-making process of distribution schemes, and ultimately, the logistics cost and carbon emission of the scheme determined by the AFO algorithm are better than the PSO algorithm and the DE algorithm. While the TS algorithm, the SS algorithm, and LINGO determine the same number of logistics centers but not the same location, although the transportation cost is similar to the AFO algorithm, the fixed cost is significantly higher than the AFO algorithm, and ultimately the AFO algorithm outperforms both the TS algorithm and the SS algorithm.

#### 5.2.2 Location scheme under demand uncertainty

**Fig 5:**
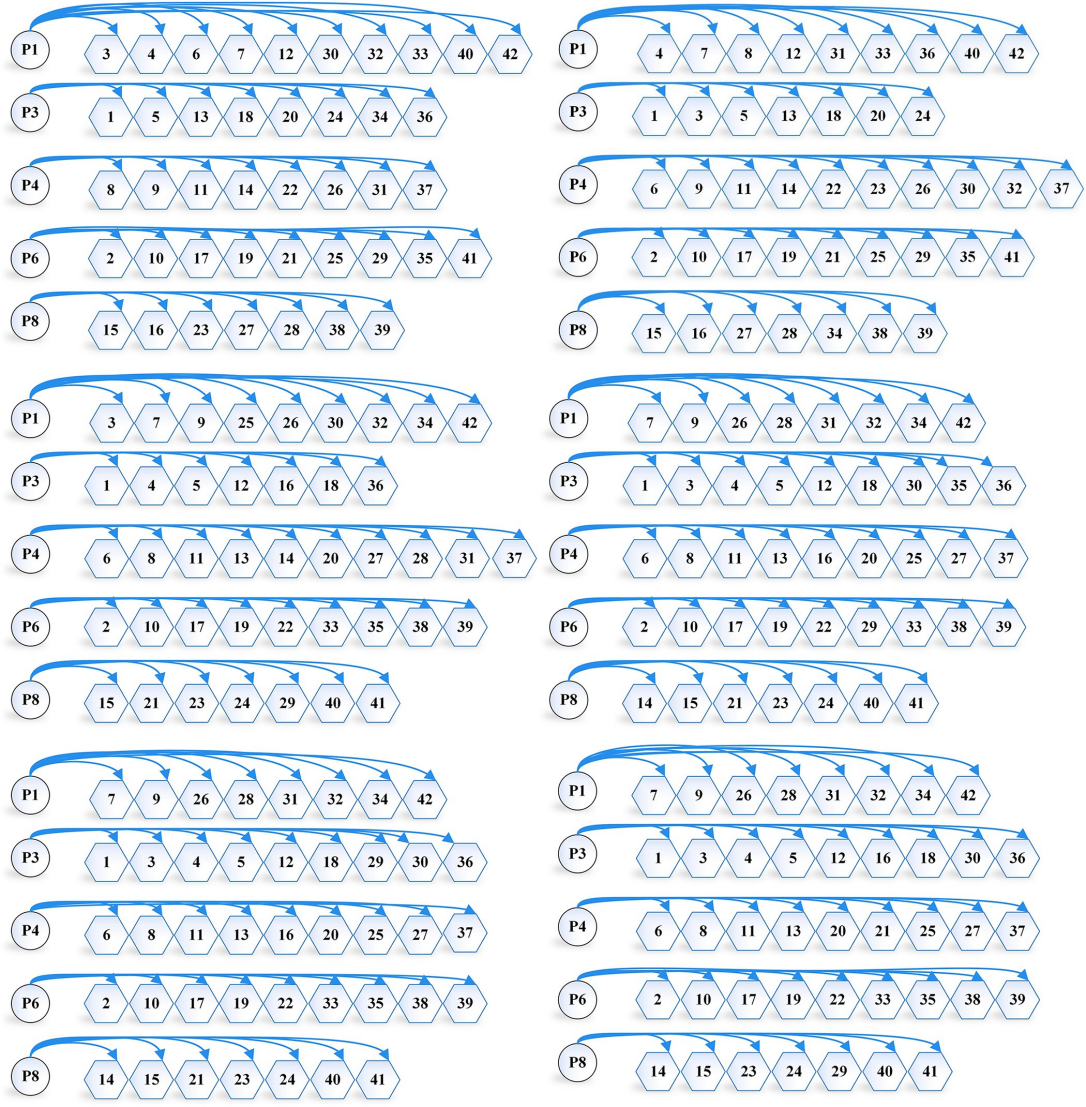
Distribution schemes under confidence level *α* = 0.9. (a) Distribution scheme determined by AFO algorithm. (b) Distribution scheme determined by PSO algorithm. (c) Distribution scheme determined by DE algorithm. (d) Distribution scheme determined by TS algorithm. (e) Distribution scheme determined by SS algorithm. (f) Distribution scheme determined by LINGO.

**Fig 6:**
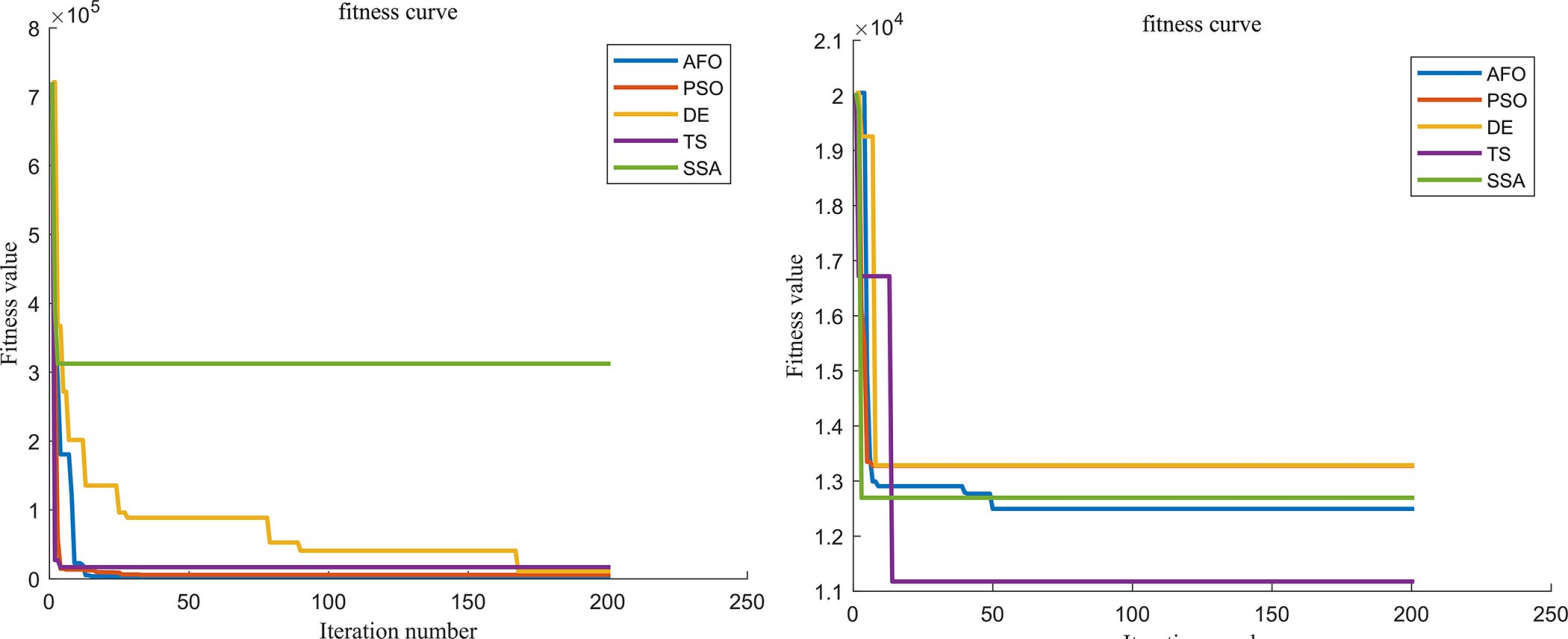
Algorithm iteration diagram. (a) Algorithm iteration diagram under demand determination. (b) Algorithm iteration diagram under confidence level *α* is 0.9.

When the confidence level is 0.9, the AFO, PSO, DE, TS, and SS algorithms, as well as LINGO, use the ideal point method to construct an evaluation function to convert the multi-objective optimization problem into a single-objective optimization problem and then use the method to solve the problem. The distribution scheme solved by the six methods is shown in Fig 5, the comparison of the solution results of the the six methods is shown in Table 7, and the algorithm iteration diagram as shown in Fig 6.

**Table 7:** Comparison of algorithm solutions at confidence level of 0.9.

Algorithm	Fixed cost (¥·10^4^)	Transport cost (¥·10^4^)	Logistic cost (¥·10^4^)	Carbon emissions (t)	Computational time(s)
AFO	329.98	342.12	672.10	104.05	4.9
PSO	329.98	342.22	672.20	104.37	2.5
DE	329.98	342.27	672.25	104.38	2.7
TS	329.98	342.40	672.38	104.74	3.7
SS	329.98	342.13	672.11	104.72	2.4
LINGO	329.98	343.64	673.62	104.78	129

From Fig 5 and Table 7, it can be found that when the confidence level *α* = 0.9, the location and number of logistics centers determined by the six methods are the same, there are differences in the distribution scheme, the AFO distribution scheme decision is better than the other five methods, and finally, the logistics cost and carbon emission of the scheme determined by the AFO algorithm are better than the other five methods.

Compared to the demand-determined case, the logistics location scheme has changed significantly. The optimum number of logistics centers is 4 under demand determination and 5 under demand uncertainty, and the distribution scheme also changes significantly. Logistics costs increase by 18.7% in the demand uncertainty case and by 23.8% in the carbon emission case compared to the demand certainty case.

By analyzing logistics location options at demand determination and confidence level *α* = 0.9, we find that the AFO algorithm has greater advantages in both determining the location of the logistics center and path planning. The AFO algorithm determines better logistics center locations compared to the TS and SS algorithms in the demand determination situation, making the global optimal solution better than the TS algorithm as well as the SS algorithm. The AFO algorithm has a stronger advantage in the path planning method when the location and number of distribution centers identified by the algorithm are the same. Compared to the remaining five methods, the AFO algorithm is able to find better transportation routes, which is the reason for the gap in the optimal solution between the AFO algorithm and the remaining five methods.

Analyzing from the perspective of computation time, the AFO algorithm’s running time during the solving process is always within 5 seconds, and although it is slower than the other four algorithms, the time difference is negligible. Compared with LINGO, the running time of the AFO algorithm is significantly shorter. This indicates that the AFO algorithm performs well in terms of operational efficiency.

In this paper, the fitness function represents the minimum distance between the solution vector and all the optimal solutions of the objective function, and the magnitude of the vector mode only indicates the distance from the ideal point in the solution space to the optimal solution of the objective function. In terms of solution results, the AFO-ideal point method algorithm outperforms the other four algorithms in terms of logistics cost and carbon emission. Therefore, the AFO-ideal point method can be used to solve the low-carbon logistics location problem.

### 5.3. Sensitivity analysis

#### 5.3.1 Freight rate sensitivity analysis

**Fig 7:**
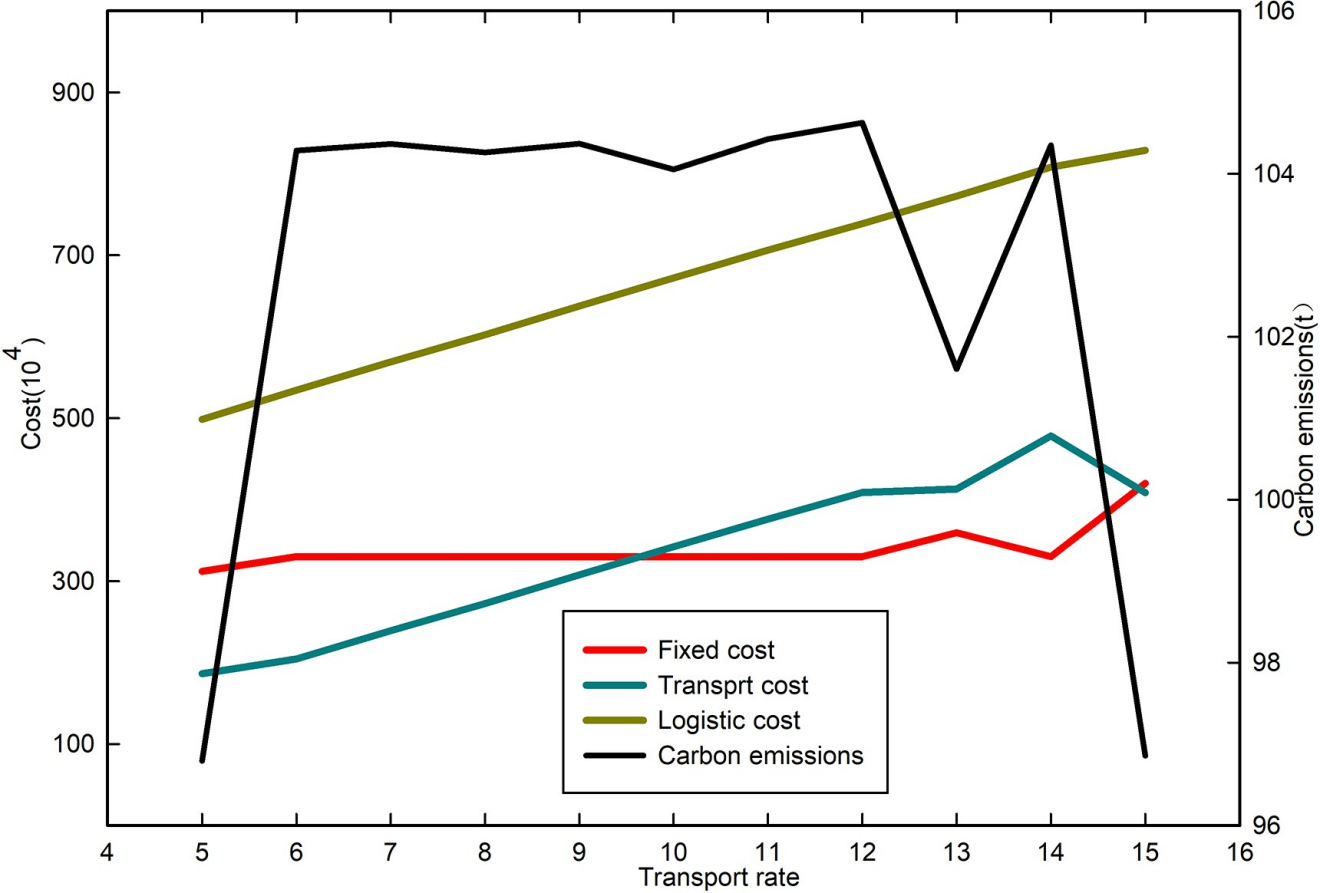
Costs and carbon emissions for different freight rates at confidence level *α* = 0.9.

When the confidence level ***α*** = 0.9, the freight rate increases from 5(¥/km∙t) to 15 (¥/km∙t), and we study the change of each cost and carbon emission, then we get the curve shown in Fig 7.

As freight costs continue to rise, overall costs are on the rise as well, but carbon emissions remain largely unchanged. The increasing cost of freight has a growing impact on site selection, and decision-makers will take different measures to reduce transportation costs. When the freight cost is between [6,12], decision-makers will adjust the distribution scheme to shorten the transportation distance and reduce transportation costs. At this time, carbon emissions fluctuate less. However, when the freight cost exceeds 12, transportation costs become the dominant factor in the logistics location problem. Decision-makers will change the location of logistics centers to reduce transportation costs and choose logistics centers that are closer to demand points but have higher fixed costs. In this case, fixed costs increase while transportation costs decrease significantly, thus reducing the upward trend of logistics costs. It is known from Eq (6) that transportation distance has a significant impact on carbon emissions, and due to the reduction in transportation distance, carbon emissions show a significant downward trend.

#### 5.3.2 Confidence level α sensitivity analysis

**Fig 8:**
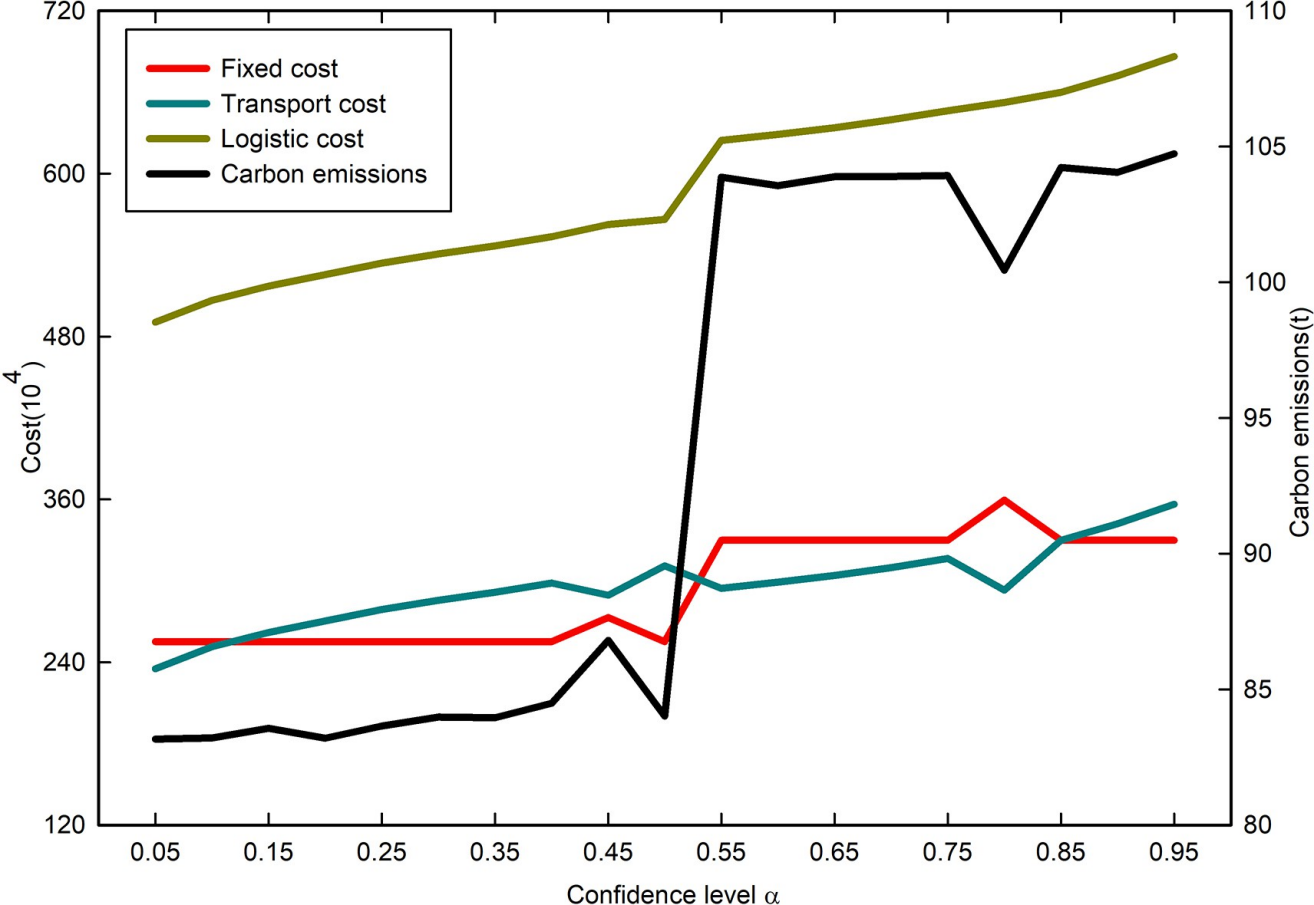
Trend of various costs and carbon emissions under different confidence level *α*.

To investigate the relationship between the location scheme under uncertainty and the confidence level ***α***, we gradually increased the value of ***α*** in increments of 0.05 and calculated the optimal location scheme that met the requirements. The results are presented in Fig 8 in the form of variation curves.

According to Fig 8, as the confidence level *α* keeps increasing, the various costs and carbon emissions gradually increase. As the confidence level *α* increases, the demand for cargo at the demand point increases, and the decision-maker needs to open more logistics centers to meet the cargo transit as well as more vehicles to meet the cargo distribution demand, which is the reason for the increasing costs and carbon emissions. From the perspective of service satisfaction, the confidence level *α* represents the percentage of demand points that can be met by the decision maker’s transportation and distribution volume. A higher confidence level means that the decision-maker can satisfy more demand. When the demand is uncertain, the decision-maker needs to pay more to satisfy all the demands at the demand points.

#### 5.3.3 Standard deviation σ_k_ sensitivity analysis

**Fig 9:**
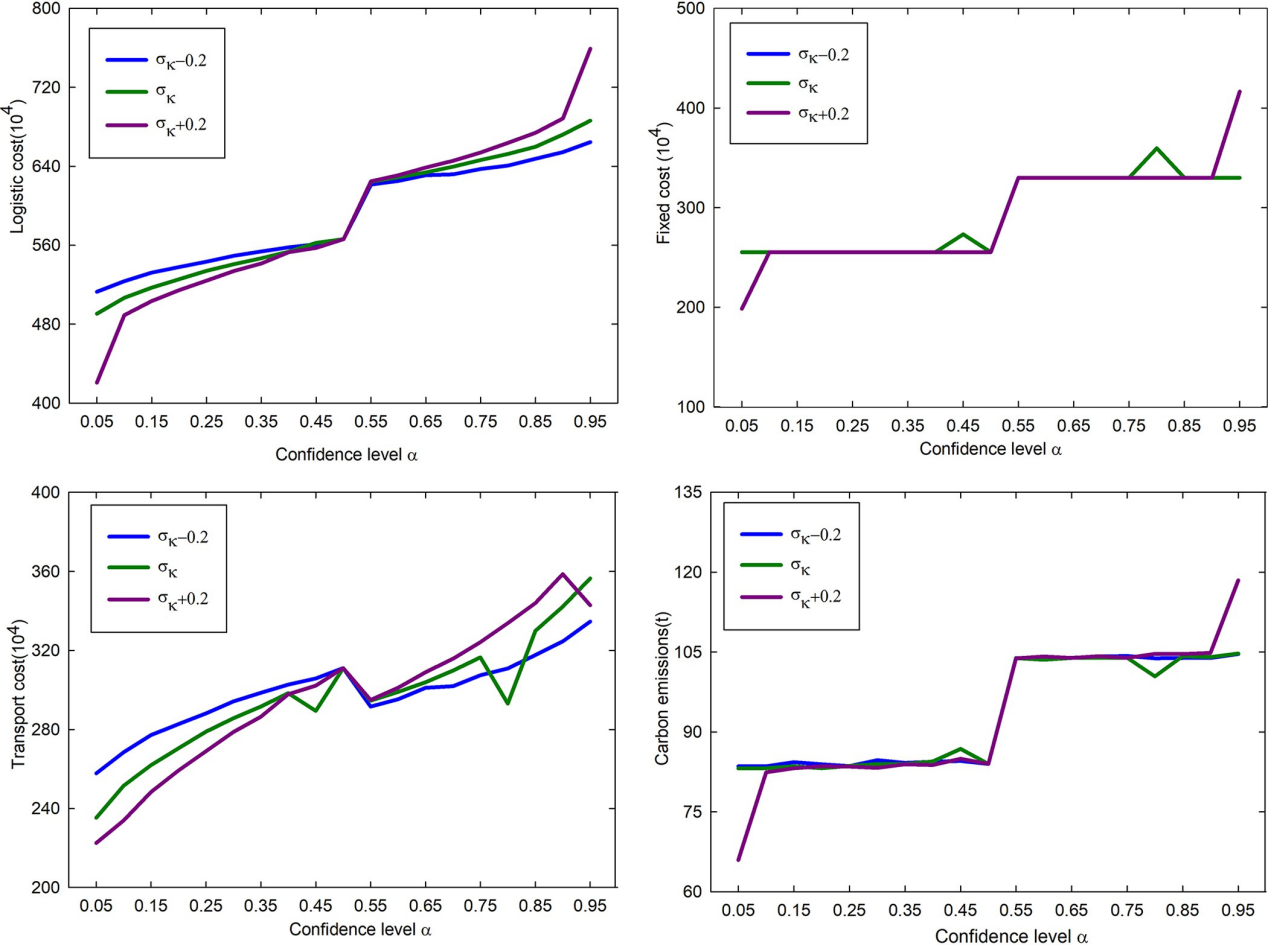
Variation curves of costs and carbon emission under different standard deviations *σ*_*k*_. (a) Logistics cost change curve under different standard deviations *σ*_*k*_. (b) Fixecd cost change curve under different standard deviations *σ*_*k*_.(c) Transport cost change curve under different standard deviations *σ*_*k*_. (d) Carbon emissions change curve under different standard deviations *σ*_*k*_.

The standard deviation is often used to assess the degree to which uncertain factors affect the outcome. In general, the larger the standard deviation, the greater the uncertainty. Therefore, this article studies the impact of the standard deviation ***σ***_***k***_ on the location scheme results while keeping the mean unchanged. To investigate this effect, we reduced and increased the standard deviation ***σ***_***k***_ of the demand point’s goods demand by 0.2 and obtained the cost and carbon emission change curves as shown in Fig 9.

According to the results in Fig 9, each cost and carbon emission increase with the increase in confidence level *α* under different*σ*_*k*_ conditions, indicating that the trend of each cost and carbon emission does not change with the increase in confidence level when the standard deviation *σ*_*k*_ increases. Under different standard deviation *σ*_*k*_ conditions, when the confidence level *α* is 0.05 and 0.95, the costs and carbon emissions appear to be more different. This is because the demand for cargo is different under different standard deviations, and the decision makers need to open different numbers of logistics centers to meet the cargo transit under different demands, and at the same time, they need to continuously adjust the distribution plan, which leads to a large difference in costs and carbon emissions. When the confidence level is between 0.45 and 0.8, the carbon emission and fixed cost appear to be more different. This is due to the change in the location selection of the logistics center, which leads to a large change in the distribution scheme and thus a very significant change in carbon emissions.

Under different standard deviation *σ*_*k*_ conditions, each cost and carbon emission are significantly different at different confidence levels, which reflects the large influence of standard deviation on the location options of logistics centers. Therefore, the influence of uncertainties on logistics location selection cannot be ignored.

In order to minimize the impact of these factors, managers can take the following measures:

1. Flexible location strategy: In the logistics location decision, a certain amount of flexibility is reserved so that the location plan can adapt to future fluctuations and changes in demand. This can reduce the risk of site selection decisions and increase the flexibility of the system.
2. Data analysis and forecasting: By analyzing historical data and forecasting demand, insight into trends and potential patterns of future demand changes can be obtained, which allows for more accurate consideration of demand uncertainty in logistics site selection decisions and more rational site selection decisions.
3. Partnership and information sharing: Establish close partnerships and information sharing mechanisms with suppliers, distributors, and partners. By sharing information and resources, we can work together to cope with demand uncertainty and improve the efficiency and adaptability of the logistics system.
4. Setting reasonable freight rates: In response to logistics site selection situations where demand is uncertain, managers should conduct a comprehensive assessment of the chosen logistics site area (including consideration of factors such as the size, frequency, and type of logistics transportation).

## 6. Discussions and conclusion

This article studies the location problem of low-carbon logistics centers under uncertain demand, using logistics cost and carbon emissions as objectives and establishing a multi-objective site selection model with random demand as a constraint using the idea of stochastic programming. Drawing on the conclusions of previous research, the demand distribution is assumed to be normal, and then the random constraints are transformed into opportunity constraints with practical significance using the idea of equivalent transformation. The AFO algorithm proposed by Yang [22] is combined with the ideal point method to solve the model, taking the location problem of a supermarket logistics center as an example to verify the effectiveness of the algorithm and the model. The optimal number and location of logistics centers under uncertain demand, as well as the optimal distribution scheme from the logistics center to the demand point, are determined to minimize logistics costs and carbon emissions. Unlike other literature on carbon emissions, this article fully considers the sources of carbon emissions from logistics centers, including the carbon emissions from transporting goods and the fixed carbon emissions from logistics centers. A relationship formula is established between transportation carbon emissions and transportation speed, cargo weight, and transportation distance, making carbon emission calculations more accurate and realistic.

The method we propose, the AFO-ideal point method, is firstly designed with an anti-premature convergence strategy to find a better global optimal solution; secondly, the ideal point method usually has a high computational efficiency in solving multi-objective planning, which can find the optimal solution in a shorter time through effective algorithms and computational techniques; the ideal point method is based on mathematical models and objective data for calculation, which reduces the influence of decision makers’ personal preferences and subjective judgments on the final results. A comparison with the other four ideal points method through example analysis verifies that the AFO-ideal point method is superior in terms of solution results, and therefore our proposed method can be applied to multi-objective low-carbon logistics location optimization problems. The practical implications and potential applications of this research are extensive, as it can be used by logistics companies or supply chain managers to make optimal site selection decisions in situations of uncertain demand, emergency relief and disaster response scenarios to achieve fast and accurate logistics decisions and efficient dispatch of supplies, and e-commerce platforms and distribution service providers to optimize their logistics networks to ensure timely delivery and maintain a competitive advantage in a highly competitive market.

By performing a sensitivity analysis of freight rates, confidence levels, and standard deviations under uncertain demand, we have obtained the following conclusions:

- Freight costs have a large impact on the results of logistics location selection. With the increase of freight costs, the impact of transportation costs on logistics locations is becoming more and more significant. In order to reduce the impact of freight costs, decision makers will optimize the logistics network by adjusting the location of logistics centers and distribution schemes so that logistics costs and carbon emissions are minimized. At the upper level, decision-makers should set a reasonable range of freight costs to achieve a low level of logistics costs and carbon emissions at the same time.
- Under demand uncertainty, the logistics cost and carbon emission will increase with the increase of confidence level *α*. This is because the confidence level *α* reflects the proportion of demand that the decision maker needs to meet at the demand point, and the total amount of cargo to be distributed will keep increasing with the increase of the confidence level *α*. When the confidence level *α* is larger, the decision maker needs to pay higher costs and generate more carbon emissions to meet the demand of cargoes at the demand point.
- The standard deviation *σ*_*k*_ has a significant impact on the site selection scheme. Under the same confidence level *α*, the difference of *σ*_*k*_ will lead to a large difference in the demand for cargo, and then decision makers need to develop different logistics centers and distribution schemes, which will lead to a large difference in costs and carbon emissions. Therefore, decision-makers need to take into account the influence of demand uncertainty factors when making logistics location decisions.

We present the limitations and scalability of the proposed method as follows.

1. This paper only considers the problem of low-carbon logistics center location with uncertain demand in the case of sufficient supply without considering the limitation of cargo supply constraints or the influence of carbon cap policies.
2. The stochastic programming method is based on the important assumption that the probability distribution of uncertain parameters is known first, while this paper assumes that the demand for goods obeys a normal distribution with reference to the results of previous studies, and in actual situations, the distribution of uncertain parameters is difficult to determine and estimate [45], which will make the research results deviate to some extent.
3. This paper does not consider the delivery time constraint of the cargo, and the demand point in real life will require the cargo to be delivered within the specified time to ensure the timeliness of the cargo.

Several research directions can be explored in the future. First, we can consider the research on the location of logistics centers with uncertain demand under the supply limitation and carbon limit policies. Specifically, we can research the supply limitation of manufacturers, the capacity limitation of transportation routes, the carbon emission limitation, and the logistics budget cost limitation. Second, when dealing with uncertain parameters, stochastic programming can be carried out in combination with robust optimization to minimize the impact of uncertain parameters. Moreover, the timeliness of the goods can be studied by transforming it into a time window penalty cost or the satisfaction of the demand point with the delivery of the cargoes.

## Supporting information

S1 Appendix AFO algorithm pseudocode. (DOCX)

S2 Appendix Demand point coordinates and demand probability distribution. (XLSX)
