# Supplementary material for: Aptenodytes forsteri optimization algorithm for low-carbon logistics network under demand uncertainty
Source: PLoS One. 2024 Jan 29;19(1):e0297223. doi: 10.1371/journal.pone.0297223 (PMC10824431; doi:10.1371/journal.pone.0297223)
Supplement: S1 Appendix — (DOCX) [file pone.0297223.s001.docx]

| AFO algorithm |
| --- |
| Input: population size N, the maximum number of iterations M, ${gap}_{0}$, $dec$,${gap}_{min}$,$\omega_{2},\omega_{4},\omega_{5}$  Output:${Xc}_{i}^{t},{Yc}_{i}^{t}$ |
| Based on uniform distribution, the position $X_{i}^{0}$ of each penguin is initialized randomly and the probability of the distribution of the i-th penguin is $r_{i}$. Calculate the temperature $Y_{i}^{t}$ for each penguin's corresponding location; Initialize each penguin’s history memory$(X_{mi}^{0},Y_{mi}^{0})$;Initialize the centre of position $(X_{ci}^{0},Y_{ci}^{0})$ of the penguin. |
| $t=1, count=0;$ |
| **while** $t\leq M$ |
| **If** $rem\left( t,gap \right)==0$ |
| Utilize strategy Ⅰ to update the $X_{c}^{t}$ |
| ${gap}_{t}=max({gap}_{min},{gap}_{t-1}-dec)$ |
| **Else**//moving strategy Ⅱ and Ⅲ |
| **For** $i=1:N$ |
| Use Eq. 1 to calculate the probability $p_{i}$ for each penguin |
| **If**  $r_{i}\geq p_{i}$ |
| Utilize strategy Ⅲ to update the $X_{i}^{t}$; |
| **Else** |
| Utilize strategy Ⅱ to update the $X_{i}^{t}$ |
| **End if** |
| Calculate the fitness $Y_{i}^{t}$ of the new position. |
| **End for** |
| **End If** |
| Update each penguin’s historical $({Xm}_{i}^{t},{Ym}_{i}^{t})$ |
| Update the centre of the penguin $({Xc}_{i}^{t},{Yc}_{i}^{t})$ |
| **If** $0.95Y_{c}^{t-1}\leq Y_{c}^{t}$ |
| count=count+1 |
| **If** $count\geq L$ |
| Carry out the strategy to prevent premature |
| **End if** |
| **End If** |
| t=t+1 |
| **End while** |
| Return the centre of the penguin $\left( X_{ci}^{t},Y_{ci}^{t} \right)$ |
